# Insights into Vincristine-Induced Peripheral Neuropathy in Aged Rats: Wallerian Degeneration, Oxidative Damage, and Alterations in ATPase Enzymes

**DOI:** 10.1021/acschemneuro.4c00342

**Published:** 2024-08-29

**Authors:** Ketlyn P. da Motta, Carolina C. Martins, Vanessa M. E. da Rocha, Mauro P. Soares, Marcia F. Mesko, Cristiane Luchese, Ethel A. Wilhelm

**Affiliations:** † Postgraduate Program in Biochemistry and Bioprospecting, Research Laboratory in Biochemical Pharmacology (LaFarBio), Center for Chemical, Pharmaceutical and Food Sciences, Federal University of Pelotas, Box 354, CEP, 96010-900 Pelotas, RS, Brazil; ‡ Regional Diagnostic Laboratory Faculty of Veterinary Medicine, Federal University of Pelotas (UFPel), CEP, 96010-900 Pelotas, RS, Brazil; § Contaminant Control Laboratory in Biomaterials (LCCBio), Federal University of Pelotas (UFPel), CEP, 96010-900 Pelotas, RS, Brazil

**Keywords:** Wallerian degeneration, vincristine, neuropathy in the old, Ca^2+^-ATPase, lipoperoxidation

## Abstract

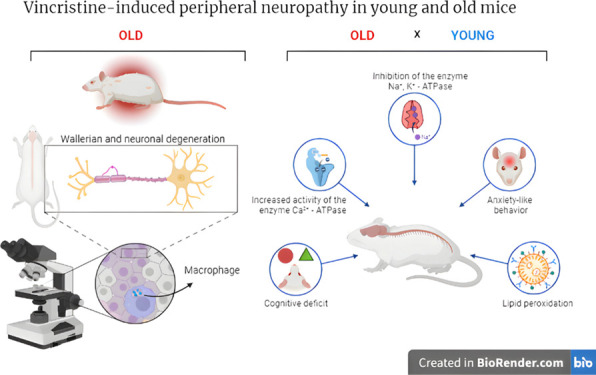

This study aimed to elucidate vincristine (VCR)-induced peripheral neuropathy in aged rats, a poorly understood neurotoxicity. Both young and old Wistar rats were administered VCR (0.1 mg/kg, intraperitoneally (i.p.)) and compared to age-matched controls (0.9% saline; 10 mg/mL, i.p.). Mechanical (MN) and thermal nociceptive (TN) responses were assessed on days 0, 6, 11, and 17. Locomotor response, cognitive ability, and anxious-like behavior were evaluated on days 14, 15, and 16. Results showed MN and TN responses in both young and old VCR-exposed rats. In old rats, VCR exacerbated MN (on days 6, 11, and 17) and TN (on days 6 and 17) responses. VCR also induced cognitive impairments and anxiety-like behavior. Histological analysis revealed Wallerian degeneration in the spinal cords of VCR-exposed rats accompanied by macrophage migration. Furthermore, VCR increased Ca^2+^-ATPase activity while inhibiting Na^+^, K^+^-ATPase activity in young and old rats. VCR altered the homeostasis of Mg^2+^-ATPase activity. Lipid peroxidation and nitrite and nitrate levels increased in young and old rats exposed to VCR. This study provides valuable insights into VCR’s mechanistic pathways in aged rats, emphasizing the need for further research in this area.

## 1 Introduction

Old age is the stage of life where diseases, pain, and neurodegenerative disorders tend to settle and develop in the body.^1,2^ Undoubtedly, pain is the most common limiting symptom in older people.^1^ Still, cancer is a frequent disease and tends to devastate the vast majority of people over 60 years of age.^3^ In this sense, three factors need to be pointed out regarding the painful state that older people with cancer face: (I) common pain already faced by the wear and tear of the aging process, (II) oncological pain caused by cancer, and (III) pain developed by chemotherapy treatment—peripheral neuropathy.^2,4,5^ Therefore, painful suffering stands out as a severe clinical condition that older people with cancer experience.

Accordingly, the chemotherapy drug vincristine (VCR) has stood out as an antineoplastic agent commonly used in clinical oncology.^6^ Despite the clinical action of VCR being focused on child cancer care, it is known that the antitumor potential of VCR is therapeutically focused on the treatment of cancers that are common in the elderly. These cancers include neuroblastomas, lymphomas (Hodgkin and non-Hodgkin), stomach adenocarcinomas, leukemias in general, and others.^7^ Unfortunately, the antitumor action of VCR is associated with the development of peripheral neuropathy.^7,8^ Peripheral neuropathy induced by VCR is one of the factors mentioned that lead to exacerbated suffering in older people with cancer. Of even more concern, VCR often develops neuropathic pain so debilitating as to promote nerve degeneration causing Wallerian degeneration.^9,10^ Wallerian degeneration developed during somatosensory injury (peripheral neuropathy) leads to impairment of anterograde and retrograde axonal transport.^4,5^ In addition, this degeneration compromises the myelin sheath of nerve cells, altering the transmission and conduction of impulses and causing cell death.^4^

Despite the scientific appeal that peripheral neuropathy caused by VCR brings about the calamity of the subject, few or rare studies have focused on exploring neurotoxicity in the elderly. Therefore, this study proposes to assist in investigating oxidative pathways, the activity of ATPase enzymes involved in the electrochemical gradient of the nervous system, and Wallerian degeneration within peripheral neuropathy induced by VCR in rats. Here, we compare the mechanisms altered by VCR in aged and young rats, aiming to elucidate the neurospecificity of actions in the elderly.

## 2 Results and Discussion

This study provides groundbreaking insight into the peripheral neuropathy process in aged rats exposed to VCR. It reveals Wallerian degeneration, neuronal damage, and macrophage migration as direct consequences of VCR exposure in the spinal cord, a novel finding. Our research also demonstrates VCR-induced mechanical and thermal nociception in young and old rats, with old rats experiencing exacerbated nociception. Notably, we observe for the first time that VCR exposure leads to cognitive and emotional impairments in both age groups. We also confirm alterations in ATPase homeostasis. We also find increased lipid peroxidation, elevated nitrite and nitrate (NO*_x_*) levels in brain tissues, and reduced nonprotein thiol (NPSH) levels in young rats exposed to VCR.

### 2.1 VCR Induces Mechanical and Thermal Nociception in Old and Young Rats

**Figure 1:**
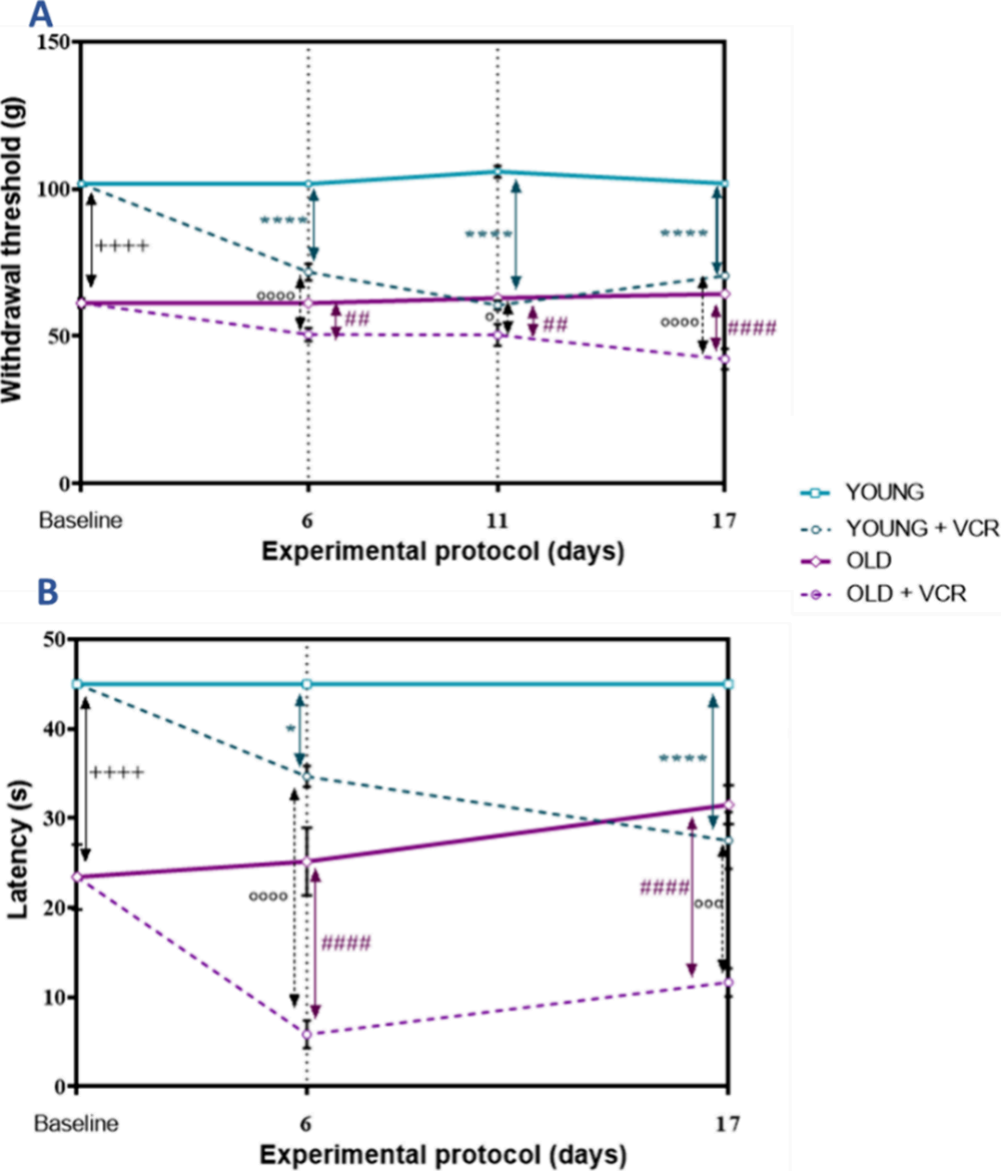
Effects of VCR sulfate (0.1 mg kg^–1^, i.p.) exposure on the paw withdrawal threshold to mechanical stimulus in the digital aesthesiometer (A) and on thermal stimulation in the hot plate test (B) in young and old rats. Each column represents the mean ± standard error of the mean (S.E.M.) of 7 animals per group. (++++) *P* < 0.0001 denotes levels of significance when comparing the young control group to the old control group; (*) *P* < 0.05 and (****) *P* < 0.0001 denote significance levels when comparing the young VCR group to the young control group; (##) *P* < 0.01 and (####) *P* < 0.0001 denote significance levels when comparing the old VCR group to the old control group; and (°) *P* < 0.05, (°°°) *P* < 0.001, and (°°°°) *P* < 0.0001 denote significance levels when comparing the old VCR group to the young VCR group. Two-way ANOVA followed by Tukey’s test was used.

VCR is widely described as triggering severe peripheral neuropathy. Young and old rats showed differences in the VCR induction profile. We found that general nociception occurred more quickly in young rats and persistently in old rats. In our study, the onset of immediate mechanical (Figure 1A) and thermal (Figure 1B) nociception was observed after the end of the last session with VCR, which persisted until day 17 of the protocol. Young and old rats exposed to VCR developed mechanical (30 and 17%, respectively) and thermal (23 and 77%) nociception on day 6 of the experimental protocol. According to the relative change in data, on day 6, VCR increased mechanical and thermal sensitivities in young rats by 0.7 and 0.8 times, respectively. Meanwhile, mechanical sensitivity increased by 0.8 times in old rats, while thermal sensitivity increased by 0.2 times. On day 11, the permanence of mechanical sensitivity (43 and 20%, respectively) was also verified in young and old rats. VCR increased mechanical and thermal sensitivities in young rats by 0.6 and 0.9 times, respectively. Similarly, both mechanical and thermal sensitivities of old rats increased by 0.8 times. The repetition of the hot plate test on day 11 was avoided to minimize the suffering of the old and young animals that were already debilitated due to exposure to VCR. On day 17 of the experimental protocol, mechanical sensitivity was verified in young (31%) and old (34%) rats that received VCR. Regarding thermal sensitivity, young (39%) and old (63%) rats retained the nociceptive response to heat. According to the relative change in data, on day 17, VCR increased the mechanical and thermal sensitivities of young rats by 0.7 and 0.6 times, respectively. In old rats, there was a 0.6 times increase in mechanical sensitivity and a 0.4 times increase in thermal sensitivity. Regarding mechanical sensitivity, old rats that received VCR also showed greater mechanical sensitivity compared to young rats that received VCR on days 6 (0.7 times), 11 (0.8 times), and 17 (0.6 times) of the experimental protocol. Still, given the data presented here, old rats exposed to VCR showed more significant thermal hypersensitivity than young rats receiving VCR on 6 (0.16 times) and 17 days (0.42 times) of behavioral evaluation.

Our study followed the development of the nociceptive condition in old rats from the last day of exposure to VCR until day 17. Mechanical presensitization was observed in old rats compared to young ones (nociception processed from the Aδ and Aβ fibers). In this regard, the linear graph of the mechanical sensitivity test shows a tendency on day 17 of the old rats to worsen the mechanical sensitization concerning the curve of the young rats, which is rising. Several authors have observed the worsening of nociceptive sensitization and consequent total loss of sensitivity due to the action of VCR, which is linked to damage to the myelin sheath and A fibers.^6,11,12^

On the other hand, young rats exposed to VCR have a greater percentage of mechanical sensitivity (day 11) than old rats. These data can be explained by the nociceptive response not compromised by degeneration in young rats exposed to VCR. That is, nondegenerated Aδ and Aβ fibers are highly activated, causing increased mechanical hyperalgesia and evoking pain. In contrast, young rats’ nerve repair capacity partially restores this exacerbation to mechanical hyperalgesia on day 17.

Along these lines, a study reported that myelinated and nonmyelinated fibers in the dorsal root ganglia, spinal cord, and supraspinal cord are responsible for pain signaling and are the most altered by aging.^2^ In this study, we verified the neuronal and axonal degeneration caused by VCR, indicating that abnormal excitability can lead to uncontrolled pain evocation and degeneration in the spinal cord. Based on VCR-induced excitability, we hypothesize that VCR exacerbates the nociceptive response to heat and causes degeneration in the old rats.^13,14^ It is also worth noting that senescence impairs nerve recovery, which can perpetuate the somatosensory damage caused by VCR.

Old rats exposed to VCR show a more significant decrease in latency to thermal stimuli than young rats with VCR. It is worth mentioning that the old rats presented a thermal presensitization about the young ones. The C fibers are unmyelinated and do not suffer the same action as the A fibers. However, the processing of C fibers involves calcium ions as a second messenger, which VCR parallelly alters.^15^ Our study unveils an elevation in Ca^2+^-ATPase activity in older rats, underscoring this as a crucial mechanism potentially contributing to the heightened thermal sensitivity observed. Consequently, the combined data from mechanical and thermal sensitivities suggest mechanisms underlying VCR effects on afferent fibers. These findings underscore that VCR-induced peripheral neuropathy can be modulated by varying mechanisms dependent on age.

### 2.2 Locomotor and Exploratory Abilities

Table 1 presents the results of the locomotor and exploratory activities of young and old rats in the open field test. Exposure to VCR and aging did not change the animals’ locomotion and exploration.

**Table 1:** Spontaneous Locomotor and Exploratory Activities of Young and Old Rats Exposed to VCR^a^

open field test					
	crossings^b^	rearings^c^		crossings^b^	rearings^c^
*young groups*			*old groups*		
control	23.2 ± 3.4	14.3 ± 1.5	control	28.3 ± 3.6	12 ± 1.0
VCR	30.8 ± 4.2	14.6 ± 2.2	VCR	21.3 ± 4.0	9 ± 1.5

a Data are reported as means ± S.E.M. for 7 animals per group. Statistical analyses were performed by two-way ANOVA, followed by the Tukey multiple comparison test when appropriate.

b Data are expressed as the number of crossings.

c Data are expressed as the number of rearings.

### 2.3 VCR-Induced Neurological Damage Can Cause Cognitive and Emotional Impairments

#### 2.3.1 Investigation of Memory and Cognition Impairments

**Figure 2:**
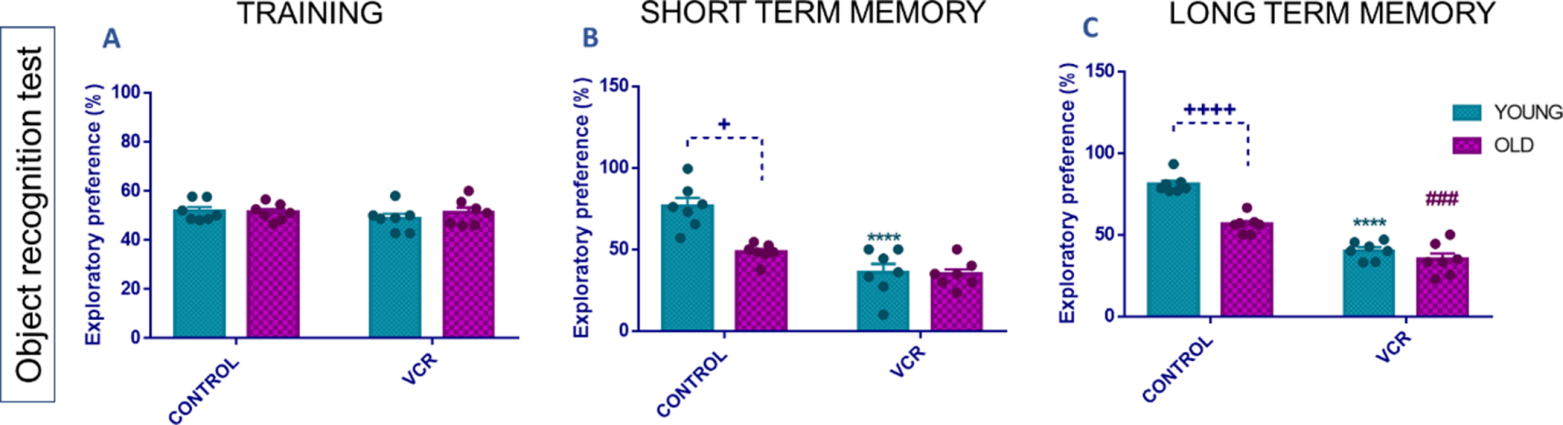
Effects of exposure to VCR sulfate (0.1 mg kg^–1^, i.p.) on the training phase (A), short-term (B), and long-term (C) memories in the object recognition test in young and old rats. Each column represents the mean ± standard error of the mean (S.E.M.) of 7 animals per group. (+) *P* < 0.05 and (++++) *P* < 0.0001 denote levels of significance when comparing the young control group to the old control group; (****) *P* < 0.0001 denotes significance levels when comparing the young VCR group to the young control group; and (###) *P* < 0.001 denotes significance levels when comparing the old VCR group to the old control group. Two-way ANOVA followed by Tukey’s test was used.

Damage to the nervous system, such as peripheral neuropathy, can damage the most diverse cognitive systems, including memory. Our study demonstrated, through the object recognition test (Figure 2), alterations in the short-term memory (STM) and long-term memory (LTM) of animals exposed to VCR. In the training phase, behavioral alterations between the experimental groups were not identified (Figure 2A). In the STM test (Figure 2B), cognitive impairment was observed only in young rats exposed to VCR compared to young control rats, with a 53% increase, indicating a 0.5 times increase in impairment. In the LTM test (Figure 2C), both young (57% increase, 0.5 times) and old (41% increase, 0.6 times) rats exposed to VCR showed impaired functionality in the transfer, evocation, and consolidation of this type of memory. It is also necessary to mention that old control rats showed cognitive impairment compared to young control rats in both STM and LTM.

Aging triggers the loss of nerve cells and the loss of neuroplasticity.^16,17^ We found that old rats previously had STM impairment, whereas VCR-induced STM impairment was found only in young ones. In LTM, both young and old rats exposed to VCR showed cognitive impairment. We found, therefore, that in LTM, VCR can affect both young and old rats in terms of memorization capacity. This finding is in line with previous studies that demonstrated the potential of VCR to destroy dentate granule cells and damage other neurons, including hippocampal pyramidal cells.^18^ Another similar study reported the neurotoxic effect of VCR on memory retention in young rats.^19^ The VCR-induced cognitive impairment in young and old rats in this study is unprecedented.

#### 2.3.2 Investigation of the Emotional Behavior

**Figure 3:**
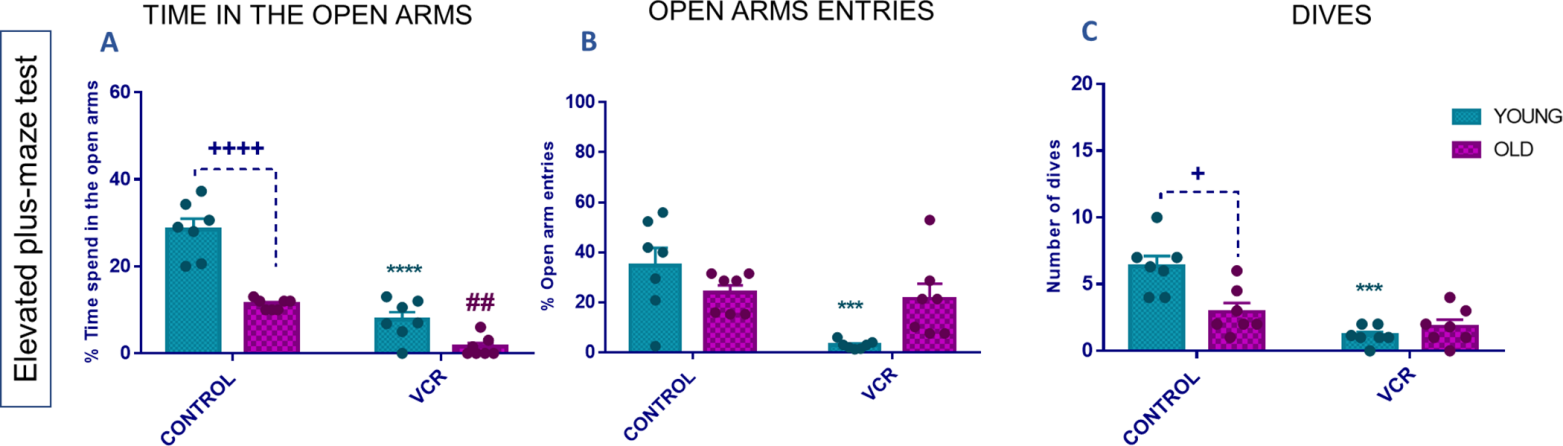
Effects of exposure to VCR sulfate (0.1 mg kg^–1^, i.p.) on the percentage of time spent in the open arms (A), percentage of open arm entries (B), and number of dips (C) in the elevated plus-maze test in young and old rats. Each column represents the mean ± standard error of the mean (S.E.M.) of 7 animals per group. (+) *P* < 0.05 and (++++) *P* < 0.0001 denote levels of significance when comparing the young control group to the old control group; (***) *P* < 0.001 and (****) *P* < 0.0001 denote significance levels when comparing the young VCR group to the young control group; and (##) *P* < 0.01 denotes significance levels when comparing the old VCR group to the old control group. Two-way ANOVA followed by Tukey’s test was used.

The elevated plus maze is validated and widely tested for assessing rodent anxious-like behavior. As shown in Figure 3A, aging per se reduced the time spent in the open arms compared to the young group; it also reduced the number of dips compared to the young group (Figure 3C). Exposure to VCR led to anxiety-like behavior in young rats, evidenced by a reduction in time spent in the open arms (Figure 3A; 82%), representing a 0.3 times decrease, a reduction in the number of entries into the open arms (Figure 3B; 90%), a 0.09 times decrease, and a reduction in the number of dives (Figure 3C; 90%), a 0.2 times decrease compared to the young control group. In old rats, VCR exposure reduced the time spent in the open arms (Figure 3A; 98%), representing a 0.1 times decrease, indicating anxiety-like behavior.

For the emotional aspect, we verified the anxiety-like behavior of young and old rats exposed to VCR. The neurotoxicity triggered by VCR leads to dangerous neuroinflammatory processes associated with increased macrophage infiltration into dorsal root ganglia and the release of cytokines and chemokines.^4,20,21^ These neuroinflammatory actions in supraspinal regions can compromise cognitive and emotional behaviors, as demonstrated in a VCR-induced neuroinflammation model with young mice.^22^ Our preclinical study is a pioneer in verifying anxiety-like behavior in old VCR-induced rats.

### 2.4 VCR Induces Neurodegeneration in the Spinal Cord of Old Rats

**Figure 4:**
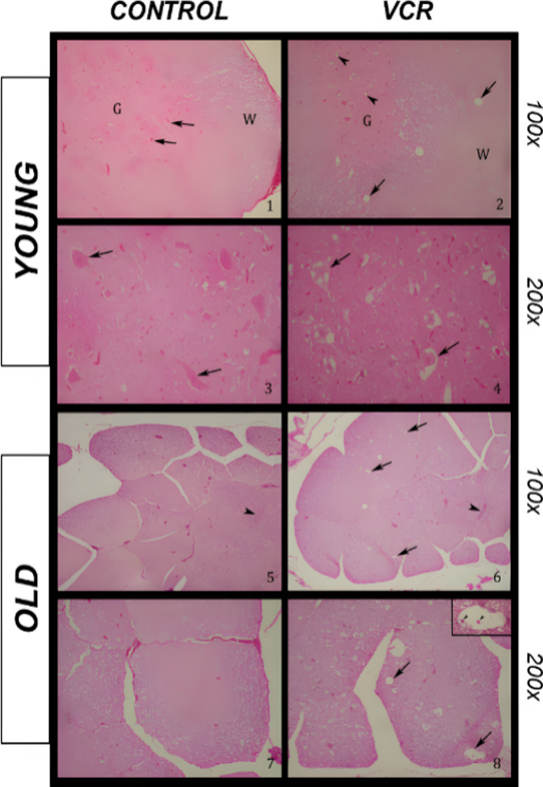
Effects of exposure to VCR sulfate (0.1 mg kg^–1^, i.p.) on histological analysis of the spinal cord of young and old rats stained with hematoxylin-eosin (HE). Blade 1: the histological profile of the lumbar spinal cord of young control rats; G denotes the gray matter, and W denotes the white matter (HE; 100X). Blade 2: the histological profile of the lumbar cord of young rats exposed to VCR; the arrow denotes large vacuoles in the white matter; the arrowhead denotes small vacuoles in the gray matter; G denotes the gray matter; and W denotes the white matter (HE; 100X). Blade 3: the histological profile of the lumbar spinal cord of young control rats; the arrow denotes motor neurons (HE; 200X). Blade 4: the histological profile of the lumbar spinal cord of young rats exposed to VCR; the arrow denotes vacuolated motor neurons (HE; 200X). Blade 5: the histological profile of the sacral spinal cord of old control rats; the arrowhead denotes the central medullary canal (HE; 100X). Blade 6: the histological profile of the sacral cord of old rats exposed to VCR; the arrow denotes white matter vacuoles; and the arrowhead denotes the central medullary canal (HE; 100X). Blade 7: the histological profile of the sacral medulla of old control rats; the image denotes lateral funiculi (HE; 200X). Blade 8: the histological profile of the sacral cord of old rats exposed to VCR; the arrow denotes white matter vacuoles; and the arrowhead image magnification (400X) denotes macrophages inside a vacuole (HE; 200X).

The control animals are represented histologically in the following images: (Figure 4; blades 1 and 3) young control group and (Figure 4; blades 5 and 7) old control group. No histological changes were observed between the control groups. The histological images of the animals submitted to VCR exposure are as follows: (Figure 4; blades 2 and 4) young VCR group and (Figure 4; blades 6 and 8) old VCR group.

The spinal cord of groups of animals subjected to VCR treatment (young and old) showed uniform development of bilateral and symmetric focally extensive vacuolation of the periaxonal space and occasional axonal spheroids. In old rats exposed to VCR, damage to the myelin sheaths was observed by the development of fragments with vacuoles of different sizes, which sometimes formed digestion chambers containing macrophages, mainly in the ventral and lateral funiculi (Figure 4; blades 6 and 8). The damage to myelin sheaths and macrophage digestion pattern was evident in aged animals exposed to VCR. In young rats exposed to VCR, the cell bodies of motor neurons were vacuolated, and spongiosis foci were present in this region’s medullary H (Figure 4; blades 2 and 4). This change was more intense in young rats exposed to VCR. Both groups submitted to VCR presented vacuolization in the white matter of the spinal cord in the lumbar and sacral segments with moderate intensity.

Indeed, the relationship between neuropathic pain and Wallerian degeneration at the peripheral level has been well reported.^23−25^ Despite this, peripheral Wallerian degeneration in old rats exposed to VCR has not yet been elucidated. Our research focuses on the deleterious effects of VCR at the central level, representing a novel aspect of our study. Previously, only the peripheral actions of VCR, contributing to neuropathy and nerve cell degeneration, had been known.^5,7,26,27^ Our study specifically targeted the spinal cord to evaluate Wallerian degeneration in a context more directly related to pain. The spinal cord was chosen for analysis because it is a critical part of the central nervous system that mediates nociceptive responses between the brain and the peripheral nervous system.^27,28^ The spinal cord is essential for processing pain and is particularly affected in chronic pain conditions.

The spinal cord plays essential roles in chronic pain, including ascending and descending pain signal transmission, pain processing through interneurons, pain perception, and descending modulation.^29,30^ Therefore, evaluation of degenerative damage in the spinal cord provides significant insights into how VCR-induced peripheral neuropathy can impact peripheral tissues and the central nervous system.

Here, histological analysis of spinal cord tissues revealed significant bilateral and symmetrical vacuolation in the periaxonal space and occasional axonal spheroids in both young and old rats exposed to VCR. Periaxonal vacuolation indicates the presence of fluid-filled spaces surrounding axons, potentially reflecting disruptions in the typical cellular environment.^31^ Axonal spheroids, on the other hand, represent rounded accumulations of the axonal material, often associated with axonal swelling, degeneration, or changes in transport processes.^32^ In summary, these histological findings induced by VCR suggest disruptions in the axonal integrity and potential disturbances in the surrounding nerve cell environment.

Notably, the damage appears more severe in old rats exposed to VCR. These rats exhibited fragments containing vacuoles of varying sizes, occasionally forming digestion chambers with macrophages, primarily in the ventral and lateral funiculi. Tissue vacuoles with macrophages suggest Wallerian degeneration, indicating ongoing phagocytic processes.^33^ The histological evidence in old rats exposed to VCR implies a neuronal body lesion, given the extensive vacuolation within neuron bodies extending to axons, ultimately leading to Wallerian degeneration. Based on the data, two scenarios can be identified: initial neuronal degeneration followed by nonclassical Wallerian degeneration resulting from neuronal injury.

Our study reveals previously unreported VCR-induced axonal damage and neuronal degeneration in the spinal cords of old rats. These data underscore how cellular apoptosis triggered by VCR can intensify in old rats, resulting in more severe neurological damage compared to their young rats. While earlier studies have documented nerve cell degeneration due to VCR in young rodents,^5,9,10^ our research illuminates the simultaneous disruption of synaptic signaling, particularly affecting pain transmission. Additionally, macrophage migration suggests that VCR may induce peripheral immune cells to release macrophages, aligning with existing research.^34^ Inflammatory macrophages, in turn, significantly influence neuroinflammation and serve as common regulators of neuropathic pain, anxiety, and depression.^35−37^

### 2.5 Imbalance Caused by VCR in the Activity of ATPases Can Compromise Neurological Activity, Causing Neuropathy and Comorbidities

#### 2.5.1 Total ATPases and Ca^2+^-ATPase Activities

ATPase enzymes are directly related to the functioning of the intracellular electrochemical gradient. Consequently, ATPases are directly related to the pathophysiology of neurodegenerative processes such as peripheral neuropathy.^38,39^

**Figure 5:**
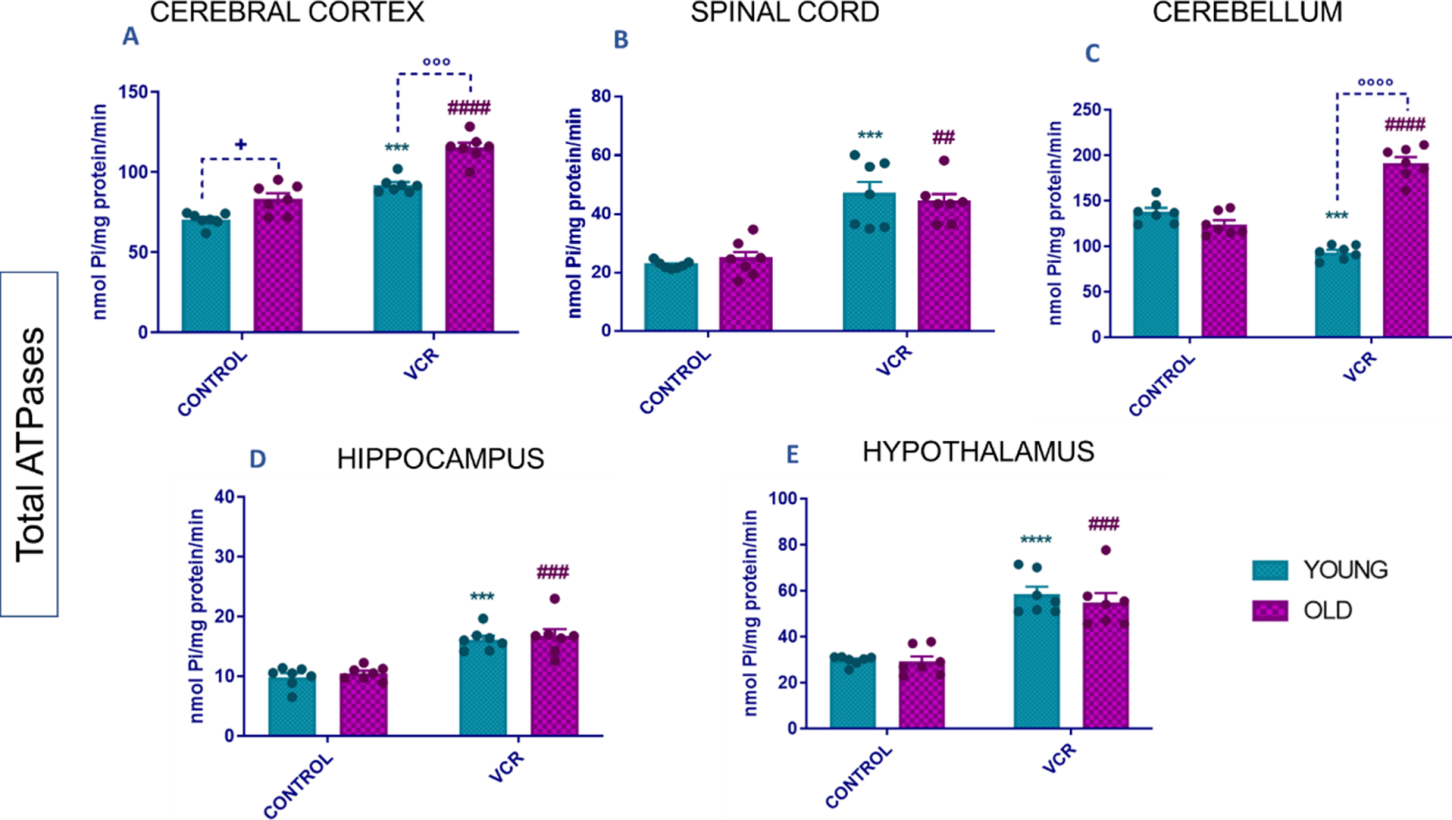
Effects of VCR sulfate (0.1 mg kg^–1^, i.p.) exposure on total ATPase activity in the cerebral cortex (A), spinal cord (B), cerebellum (C), hippocampus (D), and hypothalamus (E) of young and old rats. Each column represents the mean ± standard error of the mean (S.E.M.) of 7 animals per group. (+) *P* < 0.05 denotes levels of significance when comparing the young control group to the old control group; (***) *P* < 0.001 and (****) *P* < 0.0001 denote significance levels when comparing the young VCR group to the young control group; (##) *P* < 0.01, (###) *P* < 0.001, and (####) *P* < 0.0001 denote significance levels when comparing the old VCR group to the old control group; and (°°°) *P* < 0.001 and (°°°°) *P* < 0.0001 denote significance levels when comparing the old VCR group to the young VCR group. Two-way ANOVA followed by Tukey’s test was used.

As shown in Figure 5, we can observe the overall activity of the ATPases. The old rats per se showed an increase in total ATPase activity compared to the young group in the cerebral cortex. Exposure to VCR resulted in increased total ATPase activity in several regions of young rats compared to the young control group: cerebral cortex (Figure 5A), with a 17% increase or 1.3-fold; spinal cord (Figure 5B), with a 58% increase or 2-fold; hippocampus (Figure 5D), with a 47% increase or 1.6-fold; and hypothalamus (Figure 5E), with a 77% increase or 2-fold. However, in cerebellar tissue, total ATPase activity was inhibited in young rats exposed to VCR.

In older rats, VCR similarly led to increased total ATPase activity across various brain regions compared to the old control group: cerebral cortex (Figure 5A), with a 61% increase or 1.4-fold; spinal cord (Figure 5B), with a 124% increase or 1.8-fold; cerebellum (Figure 5A), with an 81% increase or 1.5-fold; hippocampus (Figure 5A), with an 86% increase or 1.6-fold; and hypothalamus (Figure 5A), with a 100% increase or 1.9-fold. The induction with VCR notably exacerbated the increase in total ATPase activity in older rats compared to younger rats exposed to VCR. Specifically, in the cerebral cortex, there was a 32% greater increase in ATPase activity in older rats, equating to a 1.25-fold rise. In the cerebellum, this increase was even more pronounced, with older rats showing a 107% higher activity, which is twice the level observed in younger rats.

To prove the idea that the excitability induced by VCR can trigger nerve degeneration, we verified the increase of enzymes that control the exchange of ions.^40^ Indeed, peripheral neuropathy caused by VCR is associated with increased excitability; pain, in particular, raises the levels of glutamate, the main excitatory neurotransmitter. Thus, the permanence of axonal depolarization generates the permanence of activation of nociceptors and the consequent painful sensation.^21,41^

Furthermore, the exacerbation of the activity of total ATPases in brain tissues of old rats was verified. Based on this factor, we hypothesized the possibility of worsening brain damage in these brain regions since this mechanism caused by VCR is linked to cell damage, such as impaired glial function and cell death.^4^ The cerebral cortex and cerebellum may be linked to cognitive ability. Thus, severe alterations in these regions can lead to the appearance of motor neuropathy and cognitive damage caused by VCR, as verified by our research group.^12^

**Figure 6:**
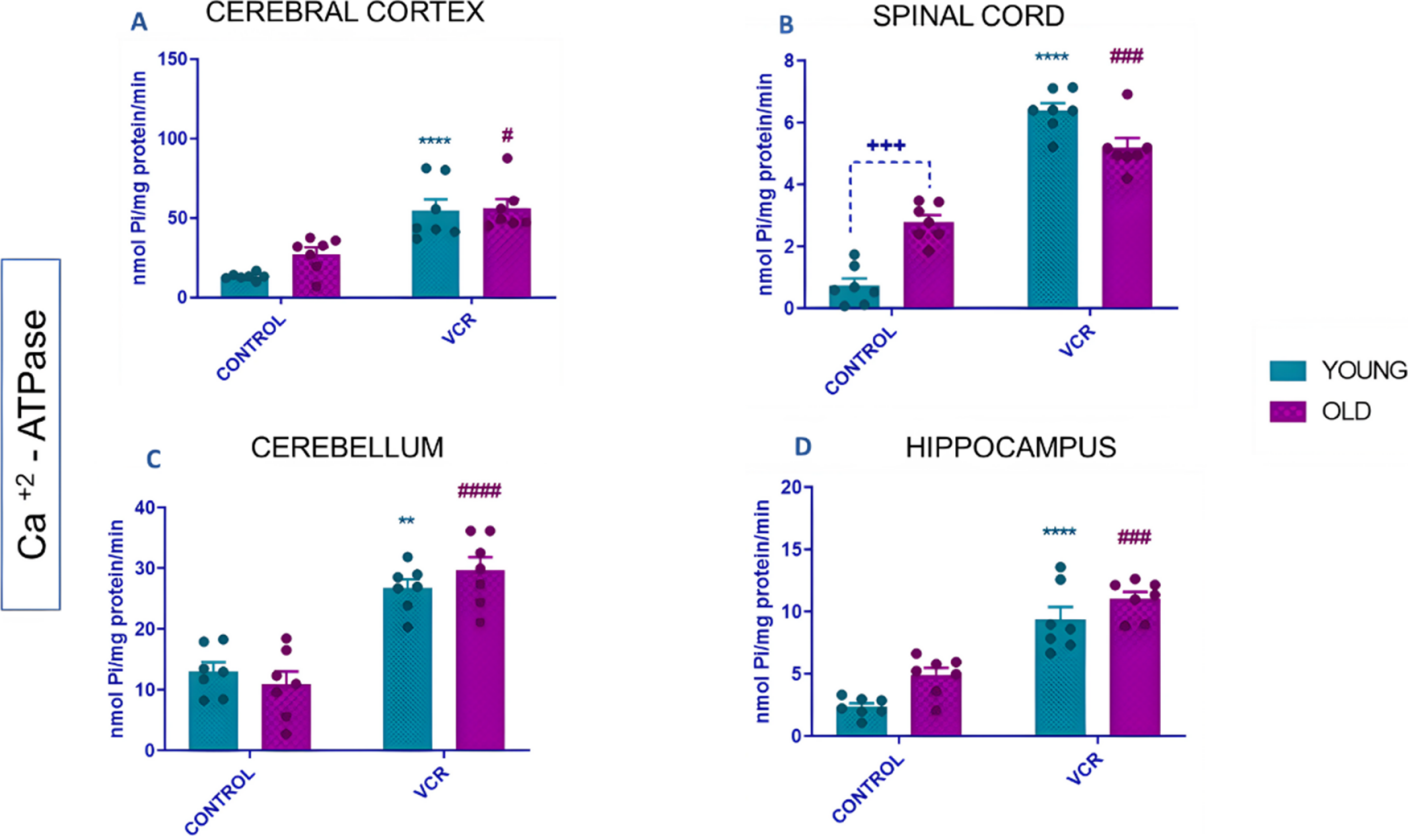
Effects of VCR sulfate (0.1 mg kg^–1^, i.p.) exposure on Ca^2+^-ATPase activity in the cerebral cortex (A), spinal cord (B), cerebellum (C), and hippocampus (D) of young and old rats. Each column represents the mean ± standard error of the mean (S.E.M.) of 7 animals per group. (+++) *P* < 0.001 denotes levels of significance when comparing the young control group to the old control group; (**) *P* < 0.01 and (****) *P* < 0.0001 denote significance levels when comparing the young VCR group to the young control group; and (#) *P* < 0.05, (###) *P* < 0.001, and (####) *P* < 0.0001 denote significance levels when comparing the old VCR group to the old control group. Two-way ANOVA followed by Tukey’s test was used.

Changes around the rate of Ca^2+^ uptake between mitochondria and the cytosolic environment are critical points in the harmful action of VCR.^4^ It was possible to verify only in the spinal cord the per se increase in Ca^2+^-ATPase activity in old rats about the group of young rats (Figure 6A). Exposure of young rats to VCR resulted in significant increases in Ca^2+^-ATPase enzyme activity compared to the young control group: in the cerebral cortex (Figure 6A), activity increased by 175% or 4.1-fold; in the spinal cord (Figure 6B), by 3000% or 8.6-fold; in the cerebellum (Figure 6C), by 145% or 2-fold; and in the hippocampus (Figure 6D), by 155% or 4-fold. In contrast, in older rats exposed to VCR, Ca^2+^-ATPase activity increased as follows: in the cerebral cortex (Figure 6A), by 141% or 2-fold; in the spinal cord (Figure 6B), by 166% or 1.8-fold; in the cerebellum (Figure 6C), by 118% or 2.7-fold; and in the hippocampus (Figure 6D), by 484% or 2.2-fold, compared to the old control group.

Our study identified the enzyme Ca^2+^-ATPase as the primary ATPase responsible for the observed outcomes in the total ATPase test. Disruptions in calcium homeostasis are recognized as a fundamental mechanism behind VCR-induced damage. When VCR operates through this mechanism, it increases calcium ions, triggering microglial activation and promoting neuroinflammatory damage, including the migration of macrophages observed in our study.^4,11,13^ Notably, VCR affects macrophage integrins, and activated integrins enhance the expression of the chemokine receptor 1 (CX3CR) motif, facilitating cell adhesion and VCR migration across the blood–brain barrier.^20^ The increased calcium levels and Ca^2+^-ATPase activity are pivotal to establishing this mechanism. Increased activity of the enzyme Ca^2+^-ATPase is presented for the first time as a common pathophysiological mechanism in young and old rats induced with VCR.

Unlike Ca^2+^-ATPase, induction with VCR inhibited the enzymatic activity of Na^+^, K^+^-ATPase from young and old rats. Such inhibition can lead to impaired sodium and potassium transport across the membrane, promoting cell swelling.^42^ Furthermore, Na^+^, K^+^-ATPase inhibitors alter neuronal firing^43,44^ and impair cognition.^45,46^ At the same time, deficiency in the Na^+^, K^+^-ATPase genes has been reported to cause memory deficits and anxiety-related behavior in mice.^47^

#### 2.5.2 Na^+^, K^+^-ATPase and Mg^2+^-ATPase Activities

**Figure 7:**
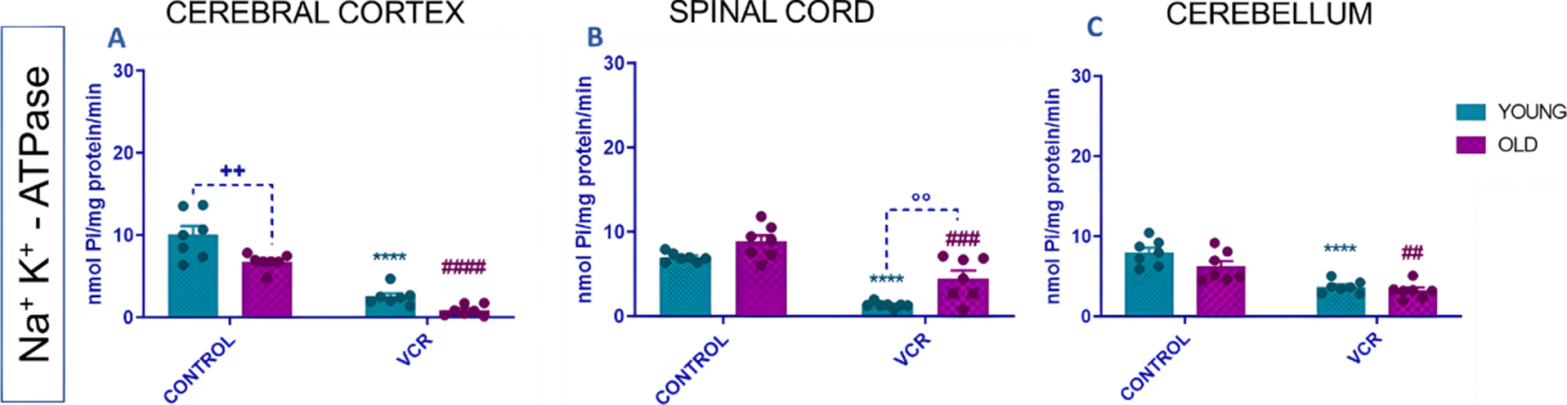
Effects of VCR sulfate (0.1 mg kg^–1^, i.p.) exposure on Na^+^, K^+^-ATPase activity in the cerebral cortex (A), spinal cord (B), and cerebellum (C) of young and old rats. Each column represents the mean ± standard error of the mean (S.E.M.) of 7 animals per group. (++) *P* < 0.01 denotes levels of significance when comparing the young control group to the old control group; (****) *P* < 0.0001 denotes significance levels when comparing the young VCR group to the young control group; (##) *P* < 0.01, (###) *P* < 0.001, and (###) *P* < 0.001 denote significance levels when comparing the old VCR group to the old control group; and (°°) *P* < 0.01 denotes significance levels when comparing the old VCR group to the young VCR group. Two-way ANOVA followed by Tukey’s test was used.

The Na^+^, K^+^-ATPase is an enzyme with crucial transmembrane activity involved in membrane depolarization and synapse functionality around the axon. Our data demonstrated that aging per se led to inhibition of the enzymatic activity of Na^+^, K^+^-ATPase only in the cerebral cortex of the young rats’ group (Figure 7A). Induction with VCR led to significant inhibition of Na^+^, K^+^-ATPase enzyme activity in young rats: in the cerebral cortex (Figure 7A), activity decreased by 90% or 0.25-fold; in the spinal cord (Figure 7B), by 86% or 0.19-fold; and in the cerebellum (Figure 7C), by 41% or 0.46-fold, compared to the young control group. In older rats exposed to VCR, Na^+^, K^+^-ATPase activity was similarly inhibited: in the cerebral cortex (Figure 7A), by 93% or 0.13-fold; in the spinal cord (Figure 7B), by 55% or 0.51-fold; and in the cerebellum (Figure 7C), by 72% or 0.52-fold, compared to the old control group. In the spinal cord (Figure 7B), there was an exacerbation in the inhibition of the Na^+^, K^+^-ATPase enzyme activity in young rats that received VCR compared to the old group that received VCR (217%).

The damage caused by VCR on Na^+^, K^+^-ATPase has not been previously reported. In neuropathic pain caused by chemotherapy drugs, Na^+^, K^+^-ATPase inhibition is supposed to be a target for pain consolidation.^44,48^ It seems that this inhibition caused by VCR in both young and old rats clarifies yet another target of action that VCR is causing. Furthermore, previous studies have suggested that dysfunctional Na^+^, K^+^-ATPase activity throughout the aging process makes neurons more susceptible to degeneration.^48,49^ In fact, the inhibition of Na^+^, K^+^-ATPase in young rats exposed to other chemotherapeutic agents has already been verified.^38,44^ However, it is hypothesized that changes in Na^+^, K^+^-ATPase activity in old rats may corroborate a greater susceptibility to neuronal damage compared to young ones.

**Figure 8:**
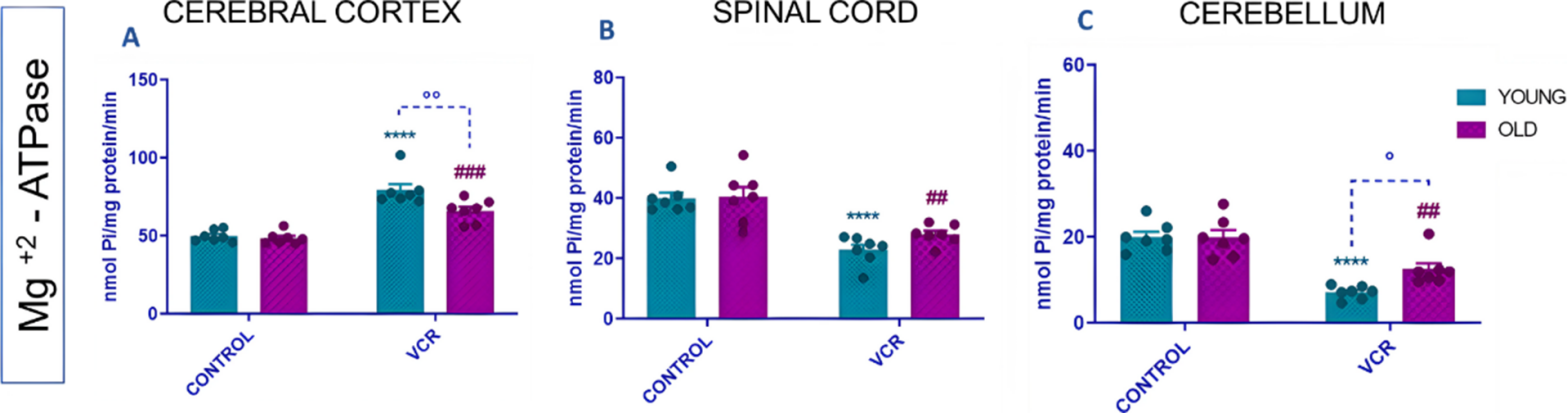
Effects of VCR sulfate (0.1 mg kg^–1^, i.p.) exposure on Mg^2+^-ATPase activity in the cerebral cortex (A), spinal cord (B), and cerebellum (C) of young and old rats. Each column represents the mean ± standard error of the mean (S.E.M.) of 7 animals per group. (****) *P* < 0.0001 denotes significance levels when comparing the young VCR group to the young control group; (##) *P* < 0.01 and (###) *P* < 0.001 denote significance levels when comparing the old VCR group to the old control group; and (°) *P* < 0.05 and (°°) *P* < 0.01 denote significance levels when comparing the old VCR group to the young VCR group. Two-way ANOVA followed by Tukey’s test was used.

Figure 8 illustrates the activity of the Mg^2+^-ATPase enzyme. Exposure of young rats to VCR resulted in increased Mg^2+^-ATPase activity in the cerebral cortex (Figure 8A), with a 69% increase or 1.4-fold, and a decrease in activity in the spinal cord (Figure 8B) and cerebellum (Figure 8C), showing a 63% or 0.6-fold inhibition and a 71% or 0.4-fold inhibition, respectively, compared to the young control rats. In older rats exposed to VCR, an increase in Mg^2+^-ATPase activity was observed in the cerebral cortex (Figure 8A), with a 36% increase or 1.3-fold. At the same time, there was inhibition of enzymatic activity in the spinal cord (Figure 8B) and cerebellum (Figure 8C), showing a 59% or 0.7-fold inhibition and a 56% or 0.6-fold inhibition, respectively, compared to the older control group.

In the cerebral cortex, the activity of the Mg^2+^-ATPase was more suppressed in old rats exposed to VCR than in young rats exposed to VCR (Figure 8A, 12%); in terms of fold change, this suppression was 0.8 times. At the same time, in the cerebellum, the enzyme activity was more suppressed in young rats exposed to VCR compared to old rats exposed to VCR (Figure 8C, 24%); in terms of fold change, this increase was 1.7 times.

Finally, changes in homeostasis in Mg^2+^-ATPase activity were observed. Mg^2+^-ATPase is the main enzyme in maintaining high intracellular brain Mg^2+^ concentrations, which is involved in the control of protein synthesis and cell growth.^42^ Based on the observation of the decrease in Mg^2+^-ATPase activity in the spinal cord and cerebellum, it is assumed that in these target tissues, VCR may be leading to impairments in the development and maintenance of new cells, which may be leading to cognitive impairments, for example. Furthermore, aging itself already leads to the loss of nerve cells, so the inhibition of Mg^2+^-ATPase activity may be a contributing factor to neurological damage.

The changes observed in Ca^2+^-ATPase, Na^+^, K^+^-ATPase, and Mg^2+^-ATPase indicate important pathophysiological mechanisms by which VCR promotes neuropathy and emotional and cognitive alterations. Briefly, changes in the homeostasis of these ATPases indicate increased cerebral excitability by Ca^2+^ flow, changes in neuronal firing and synaptic signaling, and damage in the development and maintenance of new cells.

### 2.6 Oxidative and Inflammatory Changes May Contribute to Neurodegeneration and Peripheral Neuropathy Induced by VCR

#### 2.6.1 TBARS Levels

**Figure 9:**
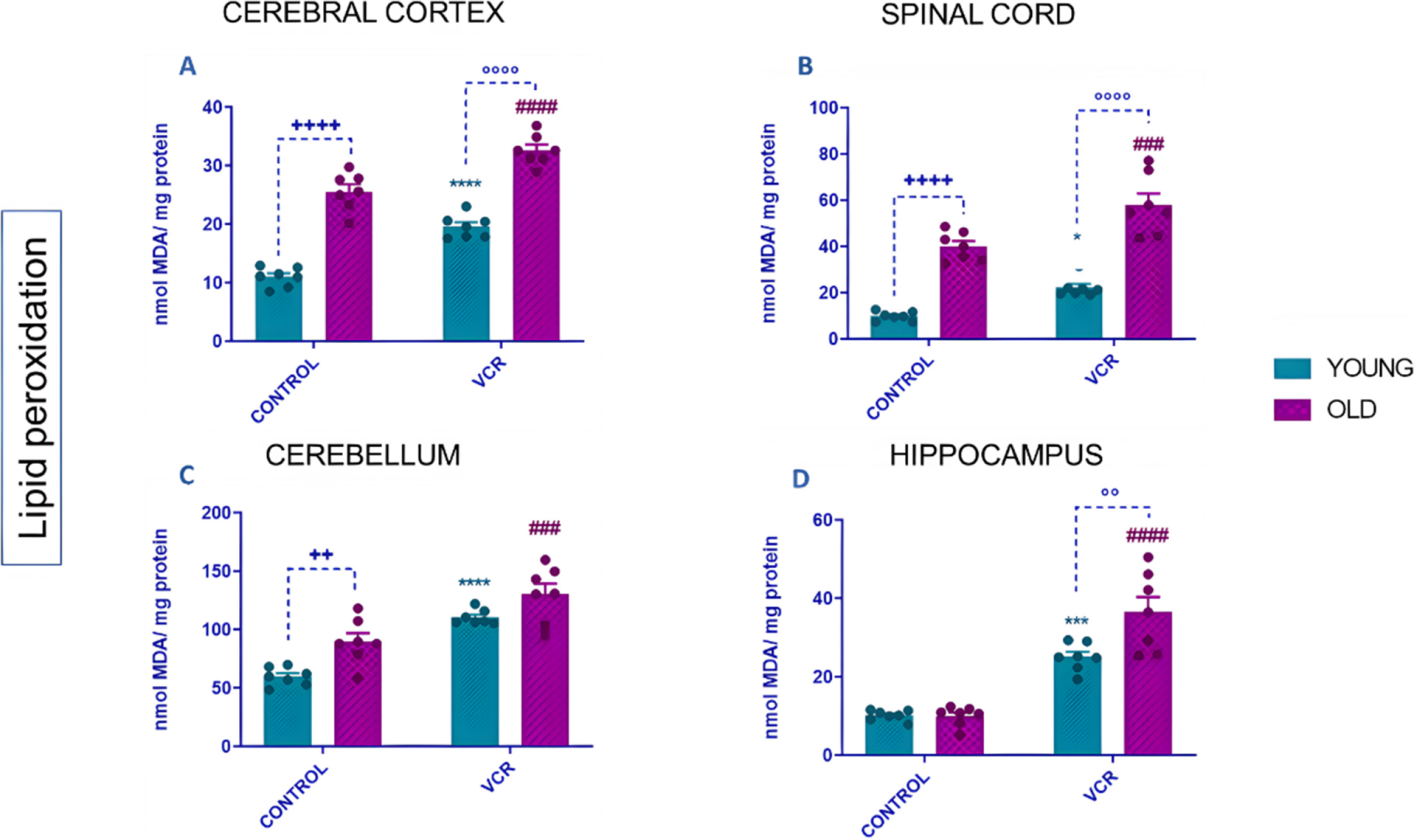
Effects of VCR sulfate (0.1 mg kg^–1^, i.p.) exposure on levels of thiobarbituric acid reactive species (TBARS) in the cerebral cortex (A), spinal cord (B), cerebellum (C), and hippocampus (D) of young and old rats. Each column represents the mean ± standard error of the mean (S.E.M.) of 7 animals per group. (++) *P* < 0.01 and (++++) *P* < 0.0001 denote levels of significance when comparing the young control group to the old control group; (*) *P* < 0.05, (***) *P* < 0.001, and (****) *P* < 0.0001 denote significance levels when comparing the young VCR group to the young control group; (###) *P* < 0.001 and (###) *P* < 0.001 denote significance levels when comparing the old VCR group to the old control group; and (°°) *P* < 0.01 and (°°°°) *P* < 0.0001 denote significance levels when comparing the old VCR group to the young VCR group. Two-way ANOVA followed by Tukey’s test was used.

Considering that chemotherapy drugs can contribute to the occurrence of oxidative damage^50^ and that the generation of lipid peroxides serves as a key marker for macromolecular oxidative injury, this study evaluated lipid peroxidation levels in brain tissues, as depicted in Figure 9.

Here, aging per se caused increased levels of lipid peroxidation in the cerebral cortex, spinal cord, and cerebellum of rats. Exposure of young rats to VCR resulted in increased lipid peroxidation in several brain regions compared to the control group of young rats: cerebral cortex (Figure 9A), with a 77% increase or 1.7-fold; spinal cord (Figure 9B), with a 105% increase or 2.2-fold; cerebellum (Figure 9C), with a 102% increase or 1.8-fold; and hippocampus (Figure 9D), with a 183% increase or 2.4-fold. In older rats, VCR also increased lipid peroxidation, although to varying extents: cerebral cortex (Figure 9A), with a 24% increase or 1.3-fold; spinal cord (Figure 9B), with a 123% increase or 1.4-fold; cerebellum (Figure 9C), with a 63% increase or 1.4-fold; and hippocampus (Figure 9D), with a 120% increase or 3.6-fold, compared to the control group of older rats. It is important to highlight that the combination of VCR and aging exacerbated the elevation of lipid peroxidation levels in the cerebral cortex (25%), spinal cord (141%), and hippocampus (33%) of these animals, in comparison to the group of young rats that were administered VCR. In terms of fold change evaluation, the data showed that an elevation of lipid peroxidation levels in the cerebral cortex was 1.6 times, that in the spinal cord was 2.6 times, and that in the hippocampus was 1.4 times in the old rats exposed to VCR when compared to young rats exposed to VCR.

The TBARS test evaluates one of the fragments of lipid peroxidation, malondialdehyde (MDA). The formation of MDA protein and MDA-acetaldehyde-protein ducts is indispensably associated with a proinflammatory reaction throughout the organism. MDA leads to the activation of Th17 lymphocytes, and MDA-acetaldehyde-protein leads to the activation of nuclear factor-κB (NFκB), influencing the activation of intercellular adhesion molecule 1 (ICAM-1) and vascular adhesion molecules (VCAM), leading directly to an increase in tumor necrosis factor-α (TNFα) expression.^51,52^

Our findings highlight increased lipid peroxidation, specifically the exacerbation of MDA fragments, as a potential trigger for the VCR-initiated inflammatory response. It is important to emphasize that this lipid damage may be directly related to the impairment of the ATPase enzymes’ activity in the phospholipid cell membrane.^40^ Our hypothesis is that such lipid damage could affect cell membrane fluidity, indirectly influencing ion exchange by altering the activity of the ATPase enzymes. Among the various tests performed, increased lipid peroxidation in old rats exposed to VCR emerges as a significant mechanism contributing to the induction of peripheral neuropathy. Lipoperoxidation can mean increased pain severity and serves as a critical contributor to neuroinflammation, as evidenced by elevated NO_*x*_ levels.

#### 2.6.2 NO_*x*_ Levels

**Figure 10:**
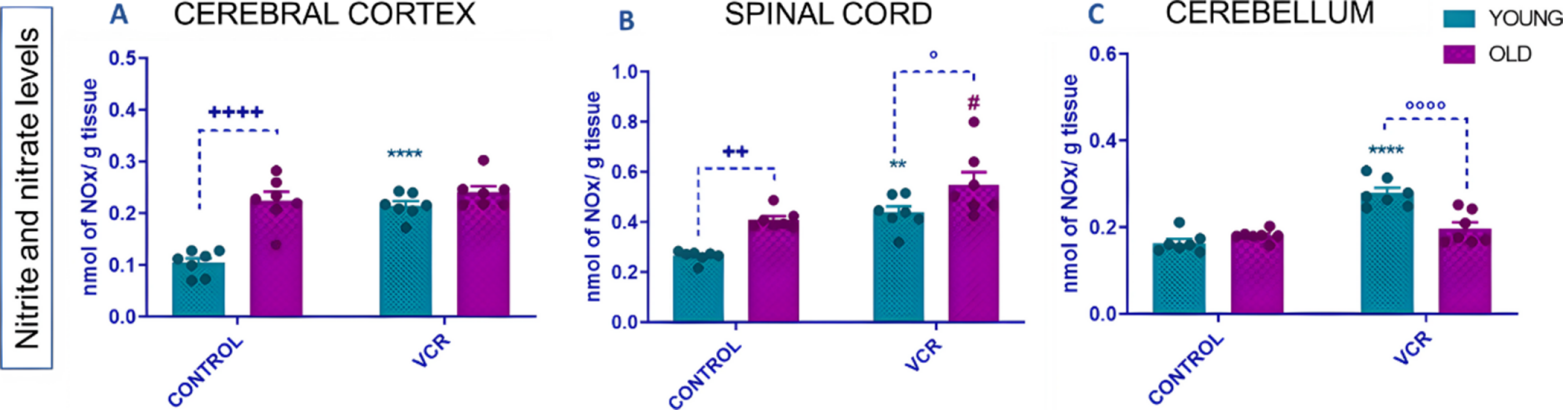
Effects of VCR sulfate (0.1 mg kg^–1^, i.p.) exposure on nitrite and nitrate levels (NO*_x_*) in the cerebral cortex (A), spinal cord (B), and cerebellum (C) of young and old rats. Each column represents the mean ± standard error of the mean (S.E.M.) of 7 animals per group. (++) *P* < 0.01 and (++++) *P* < 0.0001 denote levels of significance when comparing the young control group to the old control group; (**) *P* < 0.01 and (****) *P* < 0.0001 denote significance levels when comparing the young VCR group to the young control group; (#) *P* < 0.05 denotes significance levels when comparing the old VCR group to the old control group; and (°) *P* < 0.05 and (°°°°) *P* < 0.0001 denote significance levels when comparing the old VCR group to the young VCR group. Two-way ANOVA followed by Tukey’s test was used.

The NO*_x_* levels can be related to the formation of reactive nitrogen species and the involvement of inflammatory damage caused by nitric oxide. NO*_x_* levels in brain tissues are shown in Figure 10.

Aging per se increases NO_*x*_ levels in the cerebral cortex and spinal cord of old rats compared to those of young rats. VCR treatment increased NO*_x_* levels in the cerebral cortex (Figure 10A), rising by 143% or 2-fold compared with the control group of young rats. In the spinal cord (Figure 10B), NO*_x_* levels increased by 52% or 1.6-fold, and in the cerebellum (Figure 10C), they rose by 60% or 1.7-fold, relative to the control group of young rats. In contrast, in older rats, VCR elevated only NO_*x*_ levels in the spinal cord (Figure 10B), with a 5% increase or 1.3-fold, compared to the control group of older rats. Furthermore, NO*_x_* levels were exacerbated in the spinal cord (6%); in relationship to fold change, this increase was 1.2 times of old rats exposed to VCR compared to the young group exposed to VCR. However, in the cerebellum, there was an exacerbation of NO*_x_* levels in young rats exposed to VCR compared to the group of old ones exposed to VCR (49%), and this increase was 0.7 times.

In this study, we verified the increase of NO*_x_*, the spinal cord being a target of the VCR action, exacerbating the NO*_x_* levels in old rats. According to Kamei et al.,^53^ one of the mechanisms by which VCR triggers peripheral neuropathy is through the formation of NO*_x_*. It is noteworthy that strong evidence suggests the pro-nociceptive action of NO*_x_*. It has been reported that a NO*_x_* donor, l-arginine, inhibits voltage-gated Ca^2+^ channels in the dorsal root ganglia of cultured neurons.^54^ These results suggested that decreased NO*_x_*-cGMP pathway activity may be involved in the genesis of hyperalgesia since Ca^2+^ and Na^+^ currents are closely associated with nociception.^55,56^

#### 2.6.3 NPSH Levels

**Figure 11:**
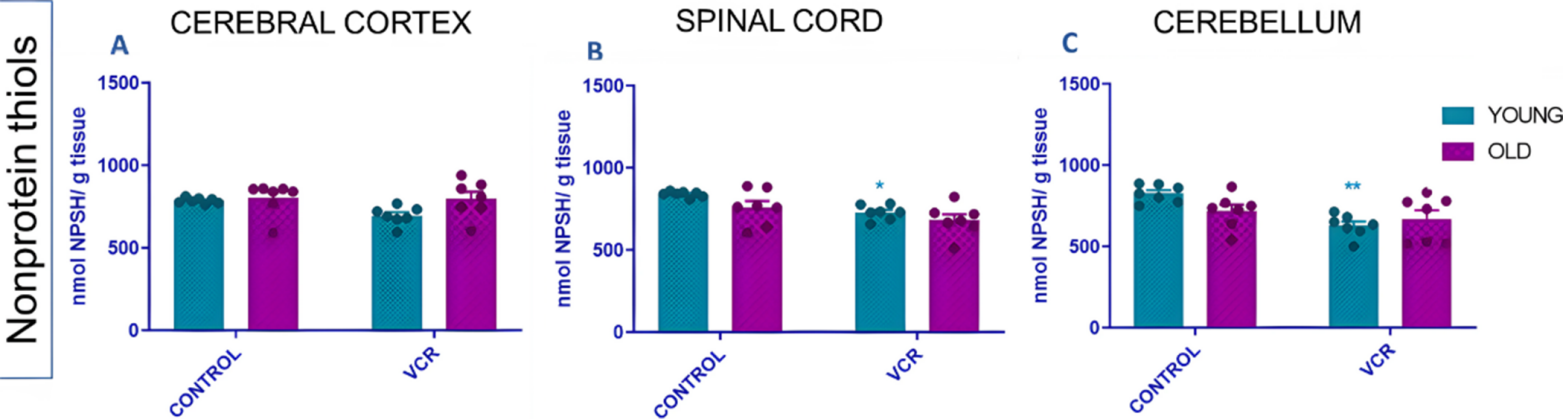
Effects of VCR sulfate (0.1 mg kg^–1^, i.p.) exposure on levels of nonprotein thiols (NPSH) in the cerebral cortex (A), spinal cord (B), and cerebellum (C) of young and old rats. Each column represents the mean ± standard error of the mean (S.E.M.) of 7 animals per group. (+) *P* < 0.05 denotes levels of significance when comparing the young control group to the old control group; (*) *P* < 0.05 and (**) *P* < 0.01 denote significance levels when comparing the young VCR group to the young control group. Two-way ANOVA followed by Tukey’s test was used.

The NPSH are markers of nonenzymatic oxidative damage and were concomitantly used for the indirect investigation of GSH, the main thiol measured in the analysis.^57^ The results of the assessment of NPSH levels can be seen in Figure 11. Exposure of young rats to VCR resulted in a significant reduction in NPSH levels in the spinal cord (Figure 11B), with a decrease of 13% or 0.86-fold, compared to the control group. Similarly, in the cerebellum (Figure 11C), NPSH levels decreased by 16% or 0.76-fold, compared to the control group of young rats. No changes in NPSH levels were observed in the cerebral cortex (Figure 11A).

We also assessed NPSH levels, specifically focusing on GSH. Young rats exposed to VCR exhibited a reduction in GSH, indicating potential damage to GSH activity, which may contribute to the persistence of VCR’s neurotoxic effects. GSH plays a crucial role in detoxifying xenobiotics in conjunction with glutathione S-transferase (GST). Hence, we propose the depletion of NPSH in the spinal cord and cerebellum of young rats exposed to VCR as a mechanism of specific oxidative damage, unrelated to senescence.

## 3 Conclusions

**Figure 12:**
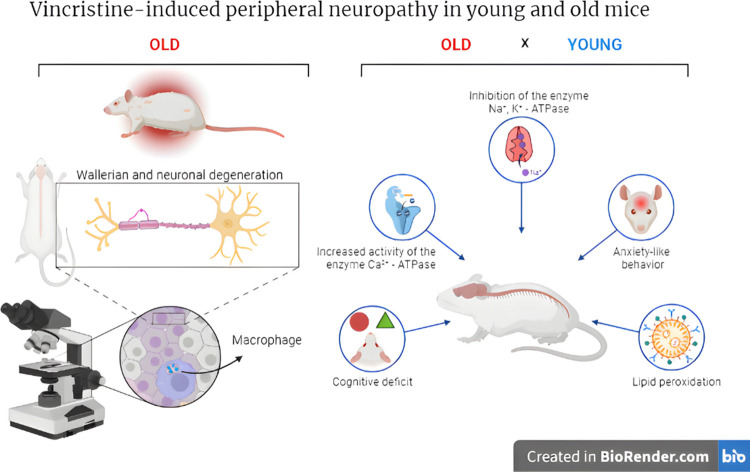
Graphical summary of neurotoxic effects induced by VCR in young and old rats. The left side of the illustration highlights the principal discovery of the study, demonstrating that neuronal and Wallerian degeneration exacerbates VCR neurotoxicity in aged rats alongside macrophage migration. On the right side, the primary mechanisms investigated in the pathophysiology of VCR-induced peripheral neuropathy in young and aged rats are delineated.

Based on the data presented in this study, the primary mechanisms arising from the neurotoxic effects of VCR in young and old rats were examined (Figure 12). The hypothesis of mechanical and thermal hypernociception in old rats exposed to VCR was substantiated for the first time. The main mechanisms potentially contributing to the somatosensory impairment caused by VCR in old rats as reported here encompass: (I) elevated lipid peroxidation in the central nervous system, (II) increased levels of NO*_x_* in the spinal cord of old rats, and (III) Wallerian degeneration and macrophage migration impacting the spinal cord in the context of VCR-associated senescence. Alterations in ATPases suggest heightened excitability, changes in neuronal firing and signaling, and disruptions in cellular maintenance. These findings imply a potential mechanism underpinning the emergence of cognitive impairment, anxiety-like behavior, and peripheral neuropathy in both young and old rats. Undoubtedly, this study advances the comprehension of VCR’s neurospecific pathways that trigger peripheral neuropathy and associated conditions. Nevertheless, further investigations are imperative to delve into VCR-induced damage, particularly concerning the aging aspect.

## 4 Materials and Methods

### 4.1 Animals

The experiments used young (2 months old) and old (16 months old) male Wistar rats. The animals were housed with free access to standard food and water; humidity (20–80%) and temperature (22 ± 2 °C) were controlled with respect to the light/dark cycle (12/12 h) of rodents. All experimental procedures were conducted following the described worldwide experimentation standards, which were recommended and previously approved by the Ethics Commission for the Use of Experimental Animals (CEUA 035767/2021-41) of the Federal University of Pelotas. Animal experiments followed the standards recommended by the Declaration of Helsinki (1964). The size, as well as the experimental number, was determined by our group using previous studies as a parameter.^44,58−60^ For euthanasia, the animals were first subjected to inhalation exposure with isoflurane, and after proof of death, decapitation was performed.

### 4.2 Drugs

VCR was acquired from INTAS Pharmaceuticals (Batch: M2103904) and allocated at 4 °C. All other chemicals used in this study were of analytical grade or obtained from Sigma-Aldrich (St. Louis, MO, USA). Rats were induced with VCR intraperitoneally (i.p.) at a constant volume of 10 mL kg^–1^ of body weight. VCR was diluted in 0.9% saline solution, which was used as a vehicle for the control groups, not submitted to chemotherapy.

### 4.3 Experimental Design

Young adult (2 months) and old (16 months) male Wistar rats were divided into four experimental groups: (I) young control, (II) young + VCR, (III) old control, and (IV) old + VCR (7 animals per group). On days 1, 3, and 5 of the experimental protocol, the animals in groups II and IV received induction with the chemotherapeutic agent VCR (0.1 mg kg^–1^, intraperitoneally), while groups I and III received a 0.9% saline solution vehicle (10 mL kg^–1^, intraperitoneally).

To investigate the induction of peripheral neuropathy, nociceptive responses were assessed: mechanical response (on days 6, 11, and 17 of the experimental protocol) was verified using the digital aesthesiometer; thermal response (on days 6 and 17 of the experimental protocol) was verified using the hot plate test. Subsequently, comorbidities associated with VCR-induced neuropathy were observed, including locomotor and exploratory abilities (on day 14), cognitive impairment (on days 14 and 15), and anxiety-like behavior (on day 16).

**Figure 13:**
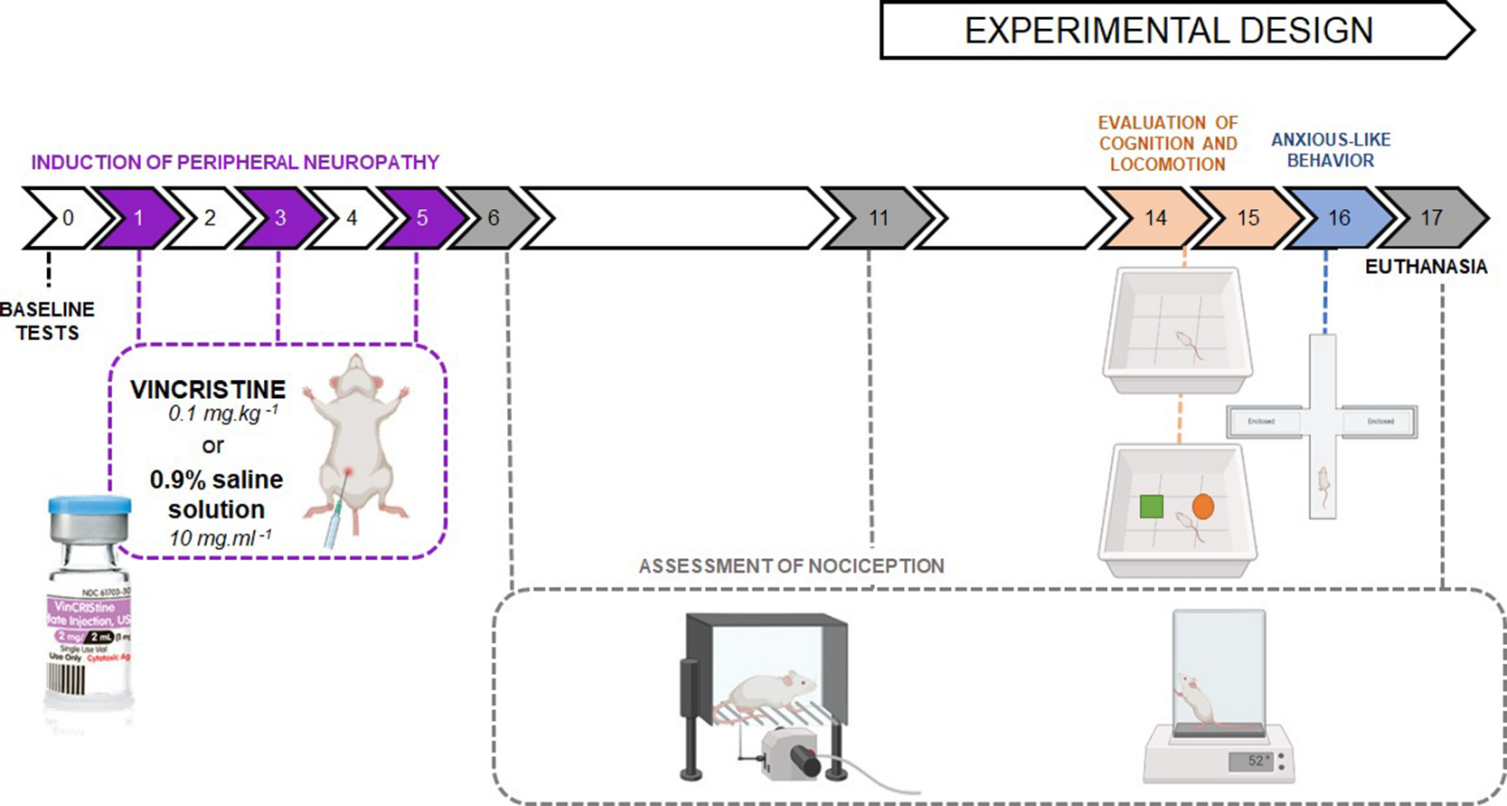
Experimental design. Young and old rats were divided into four experimental groups. On days 1, 3, and 5 of the experimental protocol, 2 groups received VCR sulfate (0.1 mg kg^–1^, intraperitoneally), while the other 2 groups received 0.9% saline solution (10 mL kg^–1^, intraperitoneally). On days 0, 6, 11, and 17, the response to mechanical hyperalgesia was verified using the digital aesthesiometer. The hot plate test verified thermal sensitivity on days 0, 6, and 17. On days 14 and 15, the locomotor and exploratory capacities of the animals were observed, followed by cognitive evaluation of the object recognition test. On day 16, the animals’ anxiety-like behavior was evaluated. Finally, on day 17 of the experimental protocol, all animals were euthanized, and nervous system structures were dissected for histological and biochemical analysis.

On day 17, the animals were euthanized by inhalation with isoflurane, and nervous system tissues related to pain processing and peripheral neuropathy were dissected for further biochemical and histological analyses. The experimental design is graphically summarized in Figure 13.

### 4.4 Evaluation of VCR-Induced Peripheral Neuropathy

#### 4.4.1 Investigation of Mechanical Hyperalgesia

Mechanical sensitivity in rats was assessed following the method described by Alamri et al.,^61^ on days 6, 11, and 17 of the experimental protocol (with some modifications). For this procedure, rats were individually allocated inside an acrylic box with wire grid floors, which was conducted in a quiet room. The test involved eliciting a hind paw flexion reflex using a hand-held force transducer (digital aesthesiometer, Insight, São Paulo, Brazil) equipped with a polypropylene tip. The withdrawal threshold of the paw was measured by applying progressive pressure perpendicular to the middle of the plantar surface of the hind paw until withdrawal occurred, with the pressure value automatically recorded. A line graph was plotted from the data generated and statistically computed to evaluate the mechanical sensitivity during the experiment. Data were expressed as the withdrawal threshold (g).

#### 4.4.2 Investigation of Thermal Hyperalgesia

Thermal sensitivity was assessed in rats following the protocol described by Woolfe and MacDonald,^62^ with some modifications. The hot plate test, a behavioral model for nociception, was utilized to investigate responses such as jumping and hind paw-licking in response to a noxious thermal stimulus. Thermal sensitivity was evaluated on days 6 and 17 of the experiment. The rats were placed in an acrylic box on a heated metal plate maintained at 52 ± 1 °C. The latency of nociceptive responses was measured. Significant decreases in paw withdrawal latency were interpreted as indicative of heat hyperalgesia. The time spent on the plate was limited to 45 s to prevent harm to the animals’ paws. A line graph was drawn from the data generated and statistically computed to evaluate the thermal sensitivity during the experiment. Data were expressed as latency (s).

### 4.5 Locomotor and Exploratory Abilities

The open field test was performed as described by Woolfe and Macdonald^62^ on day 14 of the experimental protocol. The apparatus for the open field test was made of plywood and surrounded by walls that were 30 cm high. The open field floor, 45 cm long and 45 cm wide, was divided by masking tape markers into 9 squares (3 rows of 3). In this test, each animal was placed in the center of the open field and observed for 4 min to record the locomotion (number of segments crossed with the four paws) and exploration (number of rearings with the hind limbs) of the animals. Locomotion and exploration data were expressed as the number of crossings and the number of rearings, respectively.

### 4.6 Assessment of Cognitive and Emotional Comorbidities Associated with VCR-Induced Peripheral Neuropathy

#### 4.6.1 Investigation of Memory and Cognition Impairments

The object recognition task was conducted on days 14 and 15 of the experimental protocol following the previously described method by Stangherlin et al.^63^ This behavioral test is widely employed to assess STM and LTM. On the task day (day 14 of the experimental protocol), each rat underwent a 5 min habituation session without objects to evaluate locomotor and exploratory behaviors.

Following habituation, rats underwent a training session, individually placed in the arena with two identical objects (objects A1 and A2) for 5 min. Exploration was recorded when the rat directed its nose within approximately 2 cm of the object while sniffing, touching, or looking at it. To assess STM, 1.5 h after training, rats were presented with a familiar object (A1) and a novel object (B). The exploration time was set at 5 min, enabling the measurement of learning and recognition memory. Subsequently, LTM was evaluated 24 h after training. Rats were allowed to explore a familiar object (A1) and a novel object (C) for 5 min, and the time spent exploring each object was recorded.

The objects used were positioned symmetrically within the arena. Objects A1 and A2 were identical balls, object B was a cube, and object C was a square. These objects, made of plastic material, measured 10 × 10 cm (length × height) and had distinct color patterns (blue, red, and yellow). The arena and objects were cleaned with 30% ethanol between trials to remove residues and odors. The data were expressed as percentages of exploratory preference, calculated as follows: Training = (A2/(A1 + A2)) × 100; STM = (B/(A1 + B)) × 100; LTM = (C/(A1 + C)) × 100.

#### 4.6.2 Investigation of the Emotional Behavior

The elevated plus-maze test (EPM), a validated method for measuring anxiety-like behavior in rodents, was conducted on day 16 of the experimental protocol following the procedure described by Pellow et al.^64^ The EPM apparatus consisted of two open arms (16 × 5 cm) and two closed arms (16 × 5 × 10 cm) arranged at a 90° angle, all connected to a central platform (5 × 5 cm) elevated 50 cm from the floor. Each animal was placed individually in the center of the maze, facing one of the open arms.

During a 5 min session, the frequency of entries into the open and closed arms, the time spent in the open arm, and the number of head dips (leaning toward the edge) were recorded. The data were expressed as a percentage of entries (using all four paws) and time spent in the open arms relative to the total number of entries and time in both the open and closed arms, respectively. The number of entries into the closed arms was also documented.

### 4.7 Ex Vivo Assays

#### 4.7.1 Preparation of Samples for Biochemical Analysis

Behavioral tests were instrumental in comprehending the symptomatology resulting from VCR and aging. These factors, when combined, can also induce neurospecific alterations. Consequently, biochemical assays conducted on brain and spinal cord tissues can provide further evidence of mechanistic processes affected by VCR-induced neuropathy and their association with aging.

The primary objective of the biochemical tests was to investigate axonal damage, which encompassed the disruption of the electrochemical gradient and subsequent oxidative stress. These assays encompassed the analysis of ATPase enzymes (Na^+^, K^+^-ATPase, Ca^2+^-ATPase, Mg^2+^-ATPase, and total ATPase), thiobarbituric acid reactive species (TBARS), nonprotein thiols (NPSH), and levels of nitrites and nitrates (NO*_x_*). The nervous system structures analyzed included the cerebral cortex, spinal cord, cerebellum, hippocampus, and hypothalamus. Moreover, the spinal cord was isolated for histological examination for medullary cell apoptosis analysis and Wallerian degeneration.

For biochemical analyses, samples were homogenized in 50 mM Tris-HCl buffer (pH 7.4) and subsequently centrifuged at 900 × *g* for 10 min to obtain a supernatant (S1). Specifically, for the assessment of NO*_x_* levels, samples were homogenized in 200 mM ZnSO_4_ and acetonitrile (96%), followed by centrifugation at 13,000 × *g* at 4 °C for 30 min, and the resulting S2 fraction was collected. It is worth noting that some biological tissues were not processed using all techniques due to the sample quantity not covering a sufficient volume.

#### 4.7.2 Analysis of the Medullary Histological Profile

The spine was collected and fixed by immersion in a 10% buffered formalin solution for 24 h. The spinal cord was not removed; it remained in place. The bones were decalcified in a solution of 8% hydrochloric acid and formic acid in a 1:1 ratio for an approximate period of 5 days. The samples were then cross-sectioned and routinely processed, embedded in paraffin, cut into 3–4 μm sections, stained with hematoxylin-eosin (HE), and examined under an optical microscope.

#### 4.7.3 Evaluation of the Activity of ATPase Enzymes

##### 4.7.3.1 Total ATPases and Ca^2+^-ATPase Activities

Total ATPase and Ca^2+^-ATPase activities were determined following the methodology described by Rohn et al.^65^ and adapted by Trevisan et al.^66^ Tissue samples (50 μL) were added to an incubation medium containing all the requisite substrates for the functioning of ionic pumps: 30 mM Tris-HCl (pH 7.4), 50 mM NaCl, 5 mM KCl, 6 mM MgCl_2_, 4 mM CaCl_2_, and 3 mM ATP. To correct for nonenzymatic substrate hydrolysis, control samples were prepared by adding trichloroacetic acid (TCA) after reaction termination.^66^

The activity of the Ca^2+^-ATPase enzyme was determined by subtracting the measured activity in the presence of Ca^2+^ from that determined in the absence of Ca^2+^. Meanwhile, the activity of total ATPases was assessed by analyzing the medium containing all the substrates. The color reaction was spectrophotometrically analyzed at 650 nm, and the enzymatic activities were expressed as nmol of Pi/min/mg of protein.

##### 4.7.3.2 Na^+^, K^+^-ATPase and Mg^2+^-ATPase Activities

The reaction mixture consisted of S1, 3 mM MgCl_2_, 125 mM NaCl, 20 mM KCl, and 50 mM Tris/HCl (pH 7.4), with a final volume of 500 μL. The reaction was initiated by adding ATP to a final concentration of 3.0 mM. Control samples were prepared under the same conditions with the addition of 0.1 mM ouabain. Ouabain is an inhibitor of the Na^+^, K^+^ pump, and its inclusion allowed the observation of enzyme activity related to the Mg^2+^ pump. To determine the Mg^2+^-ATPase activity, 1 mM ouabain was added to the reaction medium. The reactions were initiated by adding ATP and stopped after 30 min of incubation with the addition of 10% TCA.

The samples were further incubated at 37 °C for 30 min, and the incubation was halted by adding 10% TCA with 10 mM HgCl_2_. Enzyme activity was calculated by measuring the difference in inorganic phosphate (Pi) levels between incubations conducted in the absence and presence of ouabain. The quantification of released inorganic phosphate (Pi) was performed following the method described by Fiske and Subbarow.^67^ The color reaction mixture was spectrophotometrically analyzed at 650 nm. Results were expressed as nmol of Pi/mg protein/min.

#### 4.7.4 Involvement of Oxidative Stress

##### 4.7.4.1 TBARS Levels

TBARS levels were used as an indirect measure to assess the amount of MDA, one of the products released by lipoperoxidation. TBARS levels were measured as described by Ohkawa et al.^68^ An aliquot of S1 was added to the reaction mixture containing thiobarbituric acid (0.8%), sodium dodecyl sulfate (8.1%), and acetic acid (pH 3.4) and incubated at 95 °C for 2 h. Absorbance was measured at 532 nm with a spectrophotometer. Results were reported as nanomoles of MDA/mg of protein.

##### 4.7.4.2 NO*_x_* Levels

The nitrite and nitrate (NO_*x*_) content was measured to assess levels of reactive nitrogen species. The measurement of NO levels was performed following the method described by Miranda et al.^69^ The content of NO*_x_* was determined in a medium consisting of 2% VCl_3_ (in 5% HCl), 0.1% *N*-(1-naphthyl)ethylenediamine dihydrochloride, and 2% sulfanilamide (in 5% HCl).

After incubating at 37 °C for 60 min with an aliquot of S2, the nitrite levels were determined spectrophotometrically at 540 nm, based on the VCl_3_-mediated reduction of nitrate to nitrite. The data were expressed as nmol of NO*_x_*/g tissue.

##### 4.7.4.3 NPSH Levels

NPSH levels were assessed to directly measure oxidative damage to thiols and indirectly assess reduced glutathione (GSH) levels. The determination of NPSH levels was carried out using Ellman’s method.^70^ The supernatant was mixed with 10% TCA in a 1:1 volume ratio. The tubes were then centrifuged at 900 × *g* for 10 min, and the protein pellet was discarded. The clear supernatant was used to determine the levels of the free-thiol (SH) groups.

An aliquot of the supernatant was added to a solution containing 1 M potassium phosphate buffer (pH 7.4) and 10 mM 5,5′-dithiobis(2-nitrobenzoic acid) (DTNB). The color reaction was measured at 412 nm by using a spectrophotometer. NPSH levels were expressed as nanomoles of NPSH per gram of tissue.

#### 4.7.5 Protein Quantification

The protein concentration was measured by the method of Bradford et al.,^71^ using bovine serum albumin as the standard.

### 4.8 Statistical Analysis

The normality of the data was assessed using the D’Agostino and Pearson omnibus normality test. Statistical analysis was conducted using GraphPad Prism 7.0 software (San Diego, CA, USA). Two-way ANOVA followed by Tukey’s multiple comparisons test was employed for statistical analysis. Data were presented as mean ± standard error of the mean (S.E.M.). Main effects were reported only when the highest second-order interaction was not statistically significant. Statistical significance was defined as *P* < 0.05.

## Notes

During the preparation of this work, the authors used GPTchat (OpenAI, 2023) to improve readability and language. After using this tool, the authors have reviewed and edited the content as necessary and take full responsibility for the content of the publication.

## Abbreviations

VCR: vincristine
TBARS: thiobarbituric acid reactive species
NPSH: nonprotein thiols
NO*_x_*: nitrites and nitrates
TCA: trichloroacetic acid
GSH: reduced glutathione
CX3CR: chemokine receptor 1 motif
TNF-α: tumor necrosis factor-α
ICAM-1: intercellular adhesion molecule 1
VCAM: vascular adhesion molecules
NFκB: nuclear factor-κB
GST: glutathione S-transferase
MDA: malondialdehyde
